# LiDy(PO_3_)_4_

**DOI:** 10.1107/S1600536808016875

**Published:** 2008-06-13

**Authors:** Fathia Chehimi-Moumen, Mokhtar Férid

**Affiliations:** a Laboratoire d’Application de la Chimie aux Ressources et Substances Naturelles et à l’Environnement, Faculté des Sciences de Bizerte, 7021 Zarzouna, Tunisia; b Unité des Matériaux de Terres Rares, Centre National de Recherches en Sciences des Matériaux, BP 95, 2050 Hammam-Lif, Tunisia

## Abstract

Single crystals of lithium dysprosium polyphosphate, LiDy(PO_3_)_4_, were prepared by the flux method. The atomic arrangement is built up by infinite (PO_3_)_*n*_ chains extending along the *b* axis. Dy^3+^ and Li^+^ cations alternate in the middle of four such chains, with Dy⋯Li distances of 3.54 (1) and 3.48 (1) Å. The DyO_8_ dodeca­hedra and LiO_4_ tetra­hedra deviate significantly from the ideal geometry. Both Dy and Li occupy special positions (Wyckoff position 4*e*, site symmetry 2).

## Related literature

For related literature, see: Averbuch-Pouchot & Bagieu Beucher (1987 ▶); Ben Zarkouna *et al.* (2005 ▶; 2007 ▶); Ben Zarkouna & Driss (2004 ▶); Durif (1995 ▶); Ettis *et al.* (2006 ▶); Férid (2006 ▶); Hashimoto *et al.* (1991 ▶); Hong (1975 ▶); Horchani *et al.* (2003 ▶); Liu & Li (1983 ▶); Chehimi-Moumen & Férid (2007 ▶); Koizumi (1976 ▶); Yamada *et al.* (1974 ▶).

## Experimental

### 

#### Crystal data

LiDy(PO_3_)_4_
*M* *_r_* = 485.32
Monoclinic, 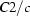

*a* = 16.269 (1) Å
*b* = 7.0236 (3) Å
*c* = 9.5781 (8) Å
β = 126.106 (3)°
*V* = 884.24 (10) Å^3^
*Z* = 4
Mo *K*α radiation
μ = 9.24 mm^−1^
*T* = 295 (2) K
0.10 × 0.09 × 0.08 mm

#### Data collection

Nonius KappaCCD diffractometer
Absorption correction: analytical (de Meulenaer & Tompa, 1965 ▶) *T*_min_ = 0.42, *T*_max_ = 0.45
3313 measured reflections
1021 independent reflections
858 reflections with *I* > 2σ(*I*)
*R*_int_ = 0.080

#### Refinement

*R*[*F* ^2^ > 2σ(*F* ^2^)] = 0.038
*wR*(*F* ^2^) = 0.087
*S* = 0.95
1021 reflections
83 parameters
Δρ_max_ = 2.26 e Å^−3^
Δρ_min_ = −2.13 e Å^−3^

### 

Data collection: *COLLECT* (Nonius, 1998 ▶); cell refinement: *DENZO*/*SCALEPACK* (Otwinowski & Minor, 1997 ▶); data reduction: *DENZO*/*SCALEPACK*; program(s) used to solve structure: *SHELXS97* (Sheldrick, 2008 ▶); program(s) used to refine structure: *SHELXL97* (Sheldrick, 2008 ▶); molecular graphics: *DIAMOND* (Brandenburg, 1999 ▶); software used to prepare material for publication: *SHELXL97*.

## Supplementary Material

Crystal structure: contains datablocks I, lidy. DOI: 10.1107/S1600536808016875/fi2064sup1.cif

Structure factors: contains datablocks I. DOI: 10.1107/S1600536808016875/fi2064Isup2.hkl

Additional supplementary materials:  crystallographic information; 3D view; checkCIF report

## Figures and Tables

**Fig. 1.**
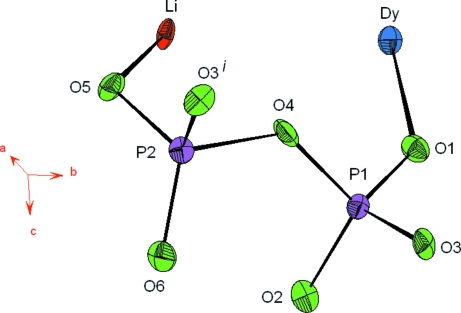
: The asymmetric unit of LiDy(PO3)4 with anisotropic displacement parameters drawn at the 50% probability level.Symmetry code: (i)-x + 1, y, -z + 1.5.

**Fig. 2.**
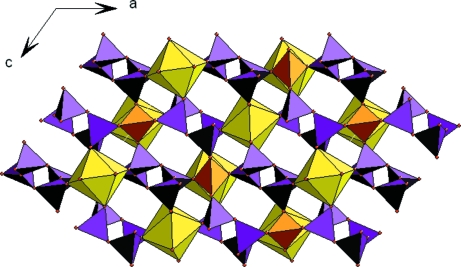
: Projection of the structure of LiDy(PO3)4 along the b axis.

**Fig. 3.**
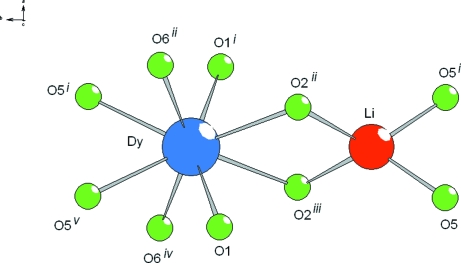
: The O-atom coordination around Dy and Li atoms showing the connection of DyO8 and LiO4 polyhedra. [Symmetry codes: (i)-x + 1, y, -z + 1.5; (ii)-x + 1, -y + 1, -z + 2; (iii)x, -y + 1, z - 1/2; (iv)x, y + 1, z; (v)-x + 1, y + 1, -z + 1.5.

**Fig. 4.**
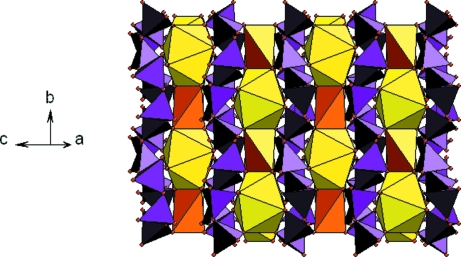
: Structural arrangement of LiDy(PO3)4 along the 1 0 1 direction.

## supplementary crystallographic information

### Comment

Condensed phosphates of rare earth and monovalent cations of general formula M^I^Ln(PO_3_)_4_ have attracted large interest in the literature of the last three decades due to their possible application as phosphors and laser materials (Yamada *et al.*, 1974; Hashimoto *et al.*, 1991; Horchani *et al.*, 2003).

In order to enrich the chemistry of this compound family, we have successfully synthesized single crystals of lithium dysprosium polyphosphate and investigated its crystal structure.

Structural studies reported for lithium lanthanide polyphosphates LiLn(PO_3_)_4_, Ln = Nd (Hong, 1975, Koizumi, 1976), Er (Liu *et al.*, 1983, Ben Zarkouna *et al.*, 2005),Yb (Ben Zarkouna *et al.*, 2004), Gd (Ettis *et al.*, 2006), Tb (Ben Zarkouna *et al.*, 2007), showed that all these compounds crystallize in space group *C*2/*c* and have similar unit-cell parameters. However, it was reported that the lithium atom is located in the (4a) site in LiNd(PO_3_)_4_ (Hong, 1975) and LiEr(PO_3_)_4_ (Liu *et al.*, 1983) and in the (4 e) site in the remaining structures. LiDy(PO_3_)_4_ is found to be isotypic with the latter group LiLn(PO_3_)_4_ previously reported. The corresponding asymmetric unit (Fig. 1) is formed by dysprosium and lithium atoms, both located in the (4 e) site, and two PO_4_ tetrahedra with all atoms in general positions.

These tetrahedra share common corners yielding infinite chains, of four tetrahedra period, extending along the 2_1_ screw axes in the b direction. Four such chains cross the unit cell (Fig. 2).

The polyphosphate chains display two type of distances, P—O terminal ranging from 1.475 (5) to 1.500 (5)Å and P—O bridging, noticeably longer, ranging from 1.573 (5) to 1.619 (5) Å. These distances are comparable with those reported for other condensed phosphates (Durif, 1995; Averbuch-Pouchot & Bagieu Beucher, 1987; Chehimi-Moumen & Férid, 2007; Férid, 2006, Ben Zarkouna *et al.*, 2007).

Dy^3+^ and Li^+^ cations lie alternatingly on the two-fold axis  in the middle of four polyphosphate chains, with Dy—Li distances of 3.55 (1) and 3.48 (1) Å. They are coordinated by eight and four external oxygen atoms, respectively. The resulting DyO_8_ dodecahedra and LiO_4_ tetrahedra are considerably distorted (Figure 3). The Dy—O and Li—O distances range from 2.288 (5) to 2.513 (5)Å and 1.95 (1) to 1.99 (1)Å respectively. The DyO_8_ dodecahedra share corners and edges with neighbouring LiO_4_ (Fig. 3) and PO_4_ tetrahedra building a three dimensional network (Fig. 4). It can be noted that, in the present arrangement, the DyO_8_ dodecahedra are isolated from each other, the shortest Dy—Dy distance is 5.563 (5) Å.

### Experimental

A mixture of Li_2_CO_3_ (2 g), Dy_2_O_3_ (0.5 g) and H_3_PO_4_ (85%, 17 ml), were mixed in a vitreous carbon crucible and preheated progressively to 473 K four 2 h. The temperature was then raised and kept at 600 K for 15 days. Colourless single crystals of LiDy(PO_3_)_4_ were isolated from the reaction mixture by washing with hot water.

### Refinement

The distances between dysprosium atoms and the highest peak and the deepest hole are respectively, 1.39 Å and 0.78 Å.

### Crystal data

**Table:**

LiDy(P_1_O_3_)_4_	*F*_000_ = 900
*M**_r_* = 485.32	*D*_x_ = 3.646 Mg m^−^^3^
Monoclinic, *C*2/*c*	Mo *K*α radiation λ = 0.71073 Å
Hall symbol: -C 2yc	Cell parameters from 1631 reflections
*a* = 16.2690 (10) Å	θ = 0.7–27.9º
*b* = 7.0236 (3) Å	µ = 9.24 mm^−^^1^
*c* = 9.5781 (8) Å	*T* = 295 (2) K
β = 126.106 (3)º	Block, colourless
*V* = 884.24 (10) Å^3^	0.10 × 0.09 × 0.08 mm
*Z* = 4	

### Data collection

**Table:**

Nonius KappaCCD diffractometer	1021 independent reflections
Radiation source: fine-focus sealed tube	858 reflections with *I* > 2σ(*I*)
Monochromator: graphite	*R*_int_ = 0.080
*T* = 295(2) K	θ_max_ = 27.7º
φ & ω scans	θ_min_ = 3.6º
Absorption correction: analytical(de Meulenaer & Tompa, 1965)	*h* = −21→20
*T*_min_ = 0.42, *T*_max_ = 0.45	*k* = −9→7
3313 measured reflections	*l* = −12→9

### Refinement

**Table:**

Refinement on *F*^2^	Primary atom site location: structure-invariant direct methods
Least-squares matrix: full	Secondary atom site location: difference Fourier map
*R*[*F*^2^ > 2σ(*F*^2^)] = 0.038	*w* = 1/[σ^2^(*F*_o_^2^) + (0.0468*P*)^2^] where *P* = (*F*_o_^2^ + 2*F*_c_^2^)/3
*wR*(*F*^2^) = 0.087	(Δ/σ)_max_ < 0.001
*S* = 0.95	Δρ_max_ = 2.26 e Å^−^^3^
1021 reflections	Δρ_min_ = −2.13 e Å^−^^3^
83 parameters	Extinction correction: none

### Special details

**Table:**

Geometry. All e.s.d.'s (except the e.s.d. in the dihedral angle between two l.s. planes) are estimated using the full covariance matrix. The cell e.s.d.'s are taken into account individually in the estimation of e.s.d.'s in distances, angles and torsion angles; correlations between e.s.d.'s in cell parameters are only used when they are defined by crystal symmetry. An approximate (isotropic) treatment of cell e.s.d.'s is used for estimating e.s.d.'s involving l.s. planes.
Refinement. Refinement of *F*^2^ against ALL reflections. The weighted *R*-factor *wR* and goodness of fit *S* are based on *F*^2^, conventional *R*-factors *R* are based on *F*, with *F* set to zero for negative *F*^2^. The threshold expression of *F*^2^ > σ(*F*^2^) is used only for calculating *R*-factors(gt) *etc*. and is not relevant to the choice of reflections for refinement. *R*-factors based on *F*^2^ are statistically about twice as large as those based on *F*, and *R*- factors based on ALL data will be even larger.

### Fractional atomic coordinates and isotropic or equivalent isotropic displacement parameters (Å^2^)

**Table:**

	*x*	*y*	*z*	*U*_iso_*/*U*_eq_	
Dy1	0.5000	0.79714 (6)	0.7500	0.01295 (18)	
P1	0.36225 (14)	0.5523 (2)	0.8849 (2)	0.0108 (4)	
P2	0.35333 (14)	0.1513 (3)	0.8039 (2)	0.0124 (4)	
O1	0.3870 (4)	0.7158 (6)	0.8178 (7)	0.0148 (11)	
O2	0.4353 (4)	0.5025 (7)	1.0727 (6)	0.0142 (10)	
O3	0.2555 (4)	0.5780 (7)	0.8535 (7)	0.0141 (11)	
O4	0.3421 (4)	0.3778 (7)	0.7655 (6)	0.0169 (11)	
O5	0.4287 (4)	0.0852 (7)	0.7730 (7)	0.0147 (10)	
O6	0.3726 (4)	0.1159 (6)	0.9727 (6)	0.0180 (12)	
Li	0.5000	0.292 (2)	0.7500	0.012 (3)	

### Atomic displacement parameters (Å^2^)

**Table:**

	*U*^11^	*U*^22^	*U*^33^	*U*^12^	*U*^13^	*U*^23^
Dy1	0.0130 (3)	0.0101 (3)	0.0150 (3)	0.000	0.0078 (2)	0.000
P1	0.0103 (9)	0.0098 (9)	0.0115 (9)	0.0003 (6)	0.0059 (8)	−0.0008 (6)
P2	0.0106 (10)	0.0115 (9)	0.0123 (9)	−0.0008 (7)	0.0053 (8)	−0.0007 (7)
O1	0.016 (3)	0.013 (3)	0.014 (3)	−0.002 (2)	0.008 (2)	0.0012 (19)
O2	0.008 (3)	0.014 (2)	0.016 (3)	−0.0022 (19)	0.004 (2)	0.001 (2)
O3	0.007 (3)	0.017 (3)	0.018 (3)	0.0056 (19)	0.007 (2)	0.006 (2)
O4	0.023 (3)	0.012 (3)	0.012 (2)	0.004 (2)	0.008 (2)	0.0057 (19)
O5	0.015 (3)	0.016 (2)	0.020 (3)	0.0014 (19)	0.014 (2)	−0.0011 (19)
O6	0.027 (3)	0.008 (2)	0.019 (3)	−0.003 (2)	0.013 (3)	−0.0008 (19)
Li	0.015 (9)	0.004 (8)	0.023 (9)	0.000	0.015 (8)	0.000

### Geometric parameters (Å, °)

**Table:**

Dy1—O6^i^	2.288 (5)	P2—O3^vi^	1.589 (5)
Dy1—O6^ii^	2.288 (5)	P2—O4	1.619 (5)
Dy1—O1	2.352 (5)	P2—Li	2.896 (5)
Dy1—O1^iii^	2.352 (5)	O2—Li^i^	1.992 (11)
Dy1—O5^iv^	2.406 (5)	O2—Dy1^i^	2.513 (5)
Dy1—O5^v^	2.406 (5)	O3—P2^vii^	1.589 (5)
Dy1—O2^ii^	2.513 (5)	O5—Li	1.951 (11)
Dy1—O2^i^	2.513 (5)	O5—Dy1^viii^	2.406 (5)
Dy1—Li^v^	3.476 (14)	O6—Dy1^i^	2.288 (5)
Dy1—Li	3.548 (14)	Li—O5^iii^	1.951 (11)
P1—O1	1.483 (5)	Li—O2^i^	1.992 (11)
P1—O2	1.500 (5)	Li—O2^ii^	1.992 (11)
P1—O4	1.573 (5)	Li—P2^iii^	2.896 (5)
P1—O3	1.589 (5)	Li—P1^i^	3.035 (5)
P1—Li^i^	3.035 (5)	Li—P1^ii^	3.035 (5)
P2—O6	1.475 (5)	Li—Dy1^viii^	3.476 (14)
P2—O5	1.495 (5)		
			
O6^i^—Dy1—O6^ii^	149.0 (2)	O3^vi^—P2—O4	100.9 (3)
O6^i^—Dy1—O1	93.73 (18)	O6—P2—Li	126.0 (2)
O6^ii^—Dy1—O1	93.71 (19)	O3^vi^—P2—Li	121.1 (2)
O6^i^—Dy1—O1^iii^	93.71 (19)	O4—P2—Li	67.6 (3)
O6^ii^—Dy1—O1^iii^	93.73 (18)	P1—O1—Dy1	139.8 (3)
O1—Dy1—O1^iii^	151.9 (2)	P1—O2—Li^i^	120.0 (4)
O6^i^—Dy1—O5^iv^	74.10 (17)	P1—O2—Dy1^i^	136.6 (3)
O6^ii^—Dy1—O5^iv^	79.93 (18)	Li^i^—O2—Dy1^i^	103.3 (3)
O1—Dy1—O5^iv^	136.63 (17)	P1—O3—P2^vii^	134.3 (4)
O1^iii^—Dy1—O5^iv^	71.43 (16)	P1—O4—P2	130.8 (3)
O6^i^—Dy1—O5^v^	79.93 (18)	P2—O5—Li	113.7 (4)
O6^ii^—Dy1—O5^v^	74.10 (17)	P2—O5—Dy1^viii^	140.9 (3)
O1—Dy1—O5^v^	71.43 (16)	Li—O5—Dy1^viii^	105.4 (3)
O1^iii^—Dy1—O5^v^	136.63 (17)	P2—O6—Dy1^i^	133.6 (3)
O5^iv^—Dy1—O5^v^	65.5 (2)	O5—Li—O5^iii^	83.7 (6)
O6^i^—Dy1—O2^ii^	137.74 (17)	O5—Li—O2^i^	119.6 (2)
O6^ii^—Dy1—O2^ii^	72.95 (15)	O5^iii^—Li—O2^i^	125.8 (2)
O1—Dy1—O2^ii^	84.22 (17)	O5—Li—O2^ii^	125.8 (2)
O1^iii^—Dy1—O2^ii^	72.18 (16)	O5^iii^—Li—O2^ii^	119.6 (2)
O5^iv^—Dy1—O2^ii^	132.43 (17)	O2^i^—Li—O2^ii^	87.2 (6)
O5^v^—Dy1—O2^ii^	137.20 (17)	O5—Li—P2	28.19 (15)
O6^i^—Dy1—O2^i^	72.95 (15)	O5^iii^—Li—P2	111.9 (5)
O6^ii^—Dy1—O2^i^	137.74 (17)	O2^i^—Li—P2	99.89 (18)
O1—Dy1—O2^i^	72.18 (16)	O2^ii^—Li—P2	108.8 (2)
O1^iii^—Dy1—O2^i^	84.22 (17)	O5—Li—P2^iii^	111.9 (5)
O5^iv^—Dy1—O2^i^	137.20 (17)	O5^iii^—Li—P2^iii^	28.19 (15)
O5^v^—Dy1—O2^i^	132.43 (17)	O2^i^—Li—P2^iii^	108.8 (2)
O2^ii^—Dy1—O2^i^	66.2 (2)	O2^ii^—Li—P2^iii^	99.89 (18)
O6^i^—Dy1—Li^v^	74.52 (12)	P2—Li—P2^iii^	140.1 (5)
O6^ii^—Dy1—Li^v^	74.52 (12)	O5—Li—P1^i^	102.86 (18)
O1—Dy1—Li^v^	104.06 (11)	O5^iii^—Li—P1^i^	108.3 (2)
O1^iii^—Dy1—Li^v^	104.06 (11)	O2^i^—Li—P1^i^	25.34 (15)
O5^iv^—Dy1—Li^v^	32.77 (12)	O2^ii^—Li—P1^i^	112.5 (5)
O5^v^—Dy1—Li^v^	32.77 (12)	P2—Li—P1^i^	92.53 (5)
O2^ii^—Dy1—Li^v^	146.88 (11)	P2^iii^—Li—P1^i^	101.64 (5)
O2^i^—Dy1—Li^v^	146.88 (11)	O5—Li—P1^ii^	108.3 (2)
O6^i^—Dy1—Li	105.48 (12)	O5^iii^—Li—P1^ii^	102.86 (18)
O6^ii^—Dy1—Li	105.48 (12)	O2^i^—Li—P1^ii^	112.5 (5)
O1—Dy1—Li	75.94 (11)	O2^ii^—Li—P1^ii^	25.34 (15)
O1^iii^—Dy1—Li	75.94 (11)	P2—Li—P1^ii^	101.64 (5)
O5^iv^—Dy1—Li	147.23 (12)	P2^iii^—Li—P1^ii^	92.53 (5)
O5^v^—Dy1—Li	147.23 (12)	P1^i^—Li—P1^ii^	137.8 (5)
O2^ii^—Dy1—Li	33.12 (11)	O5—Li—Dy1^viii^	41.9 (3)
O2^i^—Dy1—Li	33.12 (11)	O5^iii^—Li—Dy1^viii^	41.9 (3)
Li^v^—Dy1—Li	180.000 (5)	O2^i^—Li—Dy1^viii^	136.4 (3)
O1—P1—O2	118.3 (3)	O2^ii^—Li—Dy1^viii^	136.4 (3)
O1—P1—O4	106.4 (3)	P2—Li—Dy1^viii^	70.0 (3)
O2—P1—O4	111.6 (3)	P2^iii^—Li—Dy1^viii^	70.0 (3)
O1—P1—O3	112.1 (3)	P1^i^—Li—Dy1^viii^	111.1 (2)
O2—P1—O3	105.0 (3)	P1^ii^—Li—Dy1^viii^	111.1 (2)
O4—P1—O3	102.4 (3)	O5—Li—Dy1	138.1 (3)
O1—P1—Li^i^	90.9 (3)	O5^iii^—Li—Dy1	138.1 (3)
O4—P1—Li^i^	144.5 (3)	O2^i^—Li—Dy1	43.6 (3)
O3—P1—Li^i^	99.2 (2)	O2^ii^—Li—Dy1	43.6 (3)
O6—P2—O5	119.7 (3)	P2—Li—Dy1	110.0 (3)
O6—P2—O3^vi^	112.5 (3)	P2^iii^—Li—Dy1	110.0 (3)
O5—P2—O3^vi^	107.3 (3)	P1^i^—Li—Dy1	68.9 (2)
O6—P2—O4	109.7 (3)	P1^ii^—Li—Dy1	68.9 (2)
O5—P2—O4	104.9 (3)	Dy1^viii^—Li—Dy1	180.0

Symmetry codes: (i) −*x*+1, −*y*+1, −*z*+2; (ii) *x*, −*y*+1, *z*−1/2; (iii) −*x*+1, *y*, −*z*+3/2; (iv) −*x*+1, *y*+1, −*z*+3/2; (v) *x*, *y*+1, *z*; (vi) −*x*+1/2, *y*−1/2, −*z*+3/2; (vii) −*x*+1/2, *y*+1/2, −*z*+3/2; (viii) *x*, *y*−1, *z*.
